# Supplementary material for: Trypanosomes lack a canonical EJC but possess an UPF1 dependent NMD-like pathway
Source: PLoS One. 2025 Mar 7;20(3):e0315659. doi: 10.1371/journal.pone.0315659 (PMC11888146; doi:10.1371/journal.pone.0315659)
Supplement: S1A Fig — Residues that are involved in the Magoh and Y14 interaction in the human protein (pdb 2XB2) are indicated by green and blue boxes below the alignment. Residues engaging in ATP (ANP (phosphoaminophosphonic acid-adenylate ester) in the crystal structure) binding have purple boxes. DEAD-box helicase motifs (Q-motif; DEAD box) are highlighted. Hs, Homo sapiens (P38919); Ce, Caenorhabditis elegans (Q9BL61); Dm, Drosophila melanogaster (Q9VHS8); Pf, Plasmodium falciparum (Q8IKF0); Sc, Saccharomyces cerevisiae (Q12099); Tb, Trypanosoma brucei (Tb927.11.8770); Tc, Trypanosoma cruzi (C4B63_6g161). (PDF) [file pone.0315659.s004.pdf]

Figure S1A

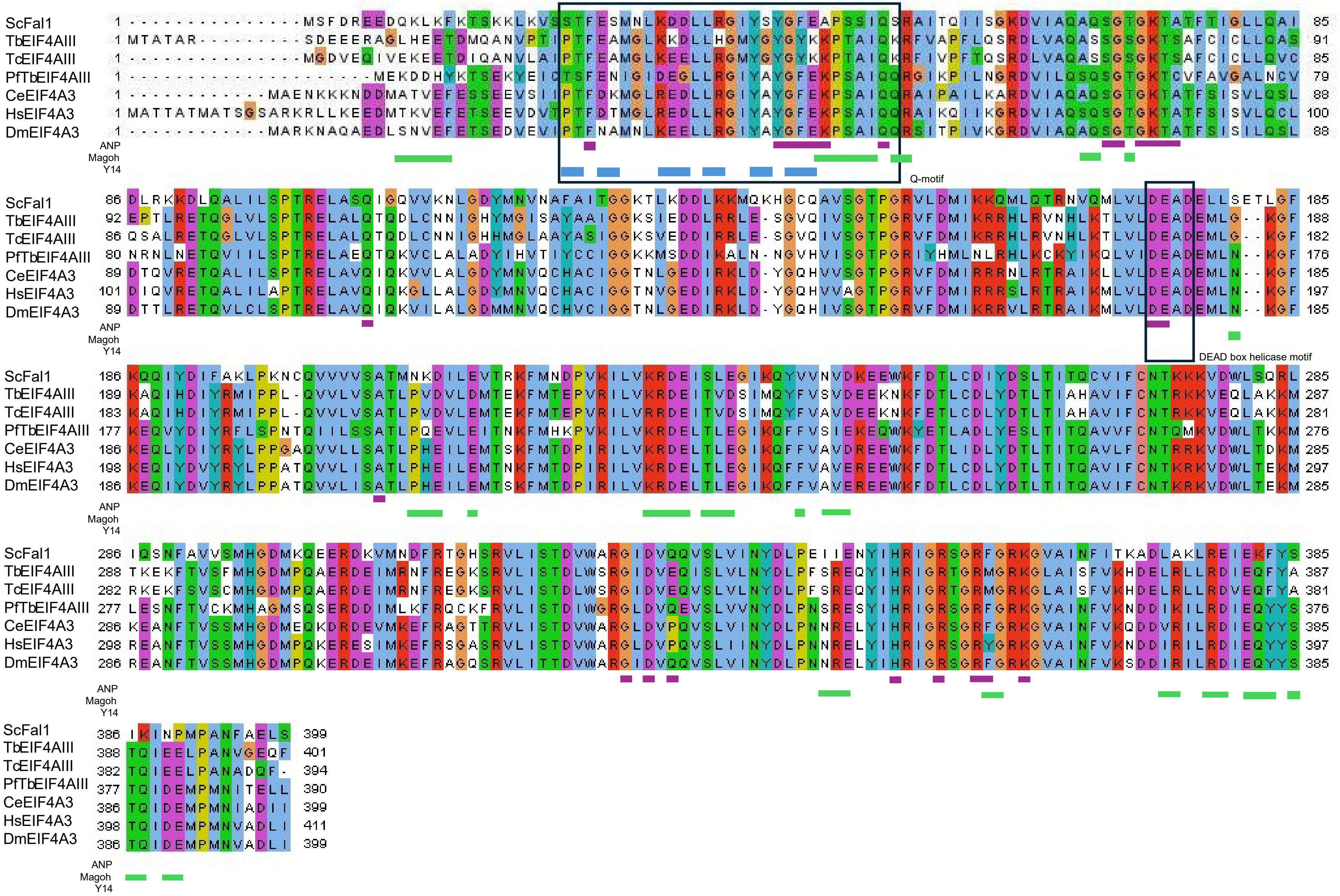

Figure S1A: eIF4AIII sequence comparison. Residues that are involved in the Magoh and Y14 interaction in the human protein (pdb 2XB2) are indicated by green and blue boxes below the alignment. Residues engaging in ATP (ANP (phosphoaminophosphonic acid-adenylate ester in the crystal structure) binding have purple boxes. DEAD-box helicase motifs (Q-motif; DEAD box) are highlighted. Hs, *Homo sapiens* (P38919); Ce, *Caenorhabditis elegans* (Q9BL61); Dm, *Drosophila melanogaster* (Q9VHS8); Pf, *Plasmodium falciparum* (Q8IKF0); Sc, *Saccharomyces cerevisiae* (Q12099); Tb, *Trypanosoma brucei* (Tb927.11.8770); Tc, *Trypanosoma cruzi* (C4B63_6g161).
