# Supplementary material for: Trypanosomes lack a canonical EJC but possess an UPF1 dependent NMD-like pathway
Source: PLoS One. 2025 Mar 7;20(3):e0315659. doi: 10.1371/journal.pone.0315659 (PMC11888146; doi:10.1371/journal.pone.0315659)
Supplement: S1B Fig — Residues that are involved in UPF2 interaction in the human protein (PMID: 19556969) are indicated (green boxes below the alignment). Residues engaging in ATP binding have purple boxes. The DEAD-box helicase motif of UPF1 is highlighted. (Hs, Homo sapiens (Q92900); Ce, Caenorhabditis elegans (O76512); Dm, Drosophila melanogaster (Q9VYS3); Eg, Euglena gracilis (EG_transcript_1144); Sc, Saccharomyces cerevisiae (P30771); Tb, Trypanosoma brucei (Tb927.5.2140); Tc, Trypanosoma cruzi (C3747_24g302). (PDF) [file pone.0315659.s005.pdf]

Figure S1B

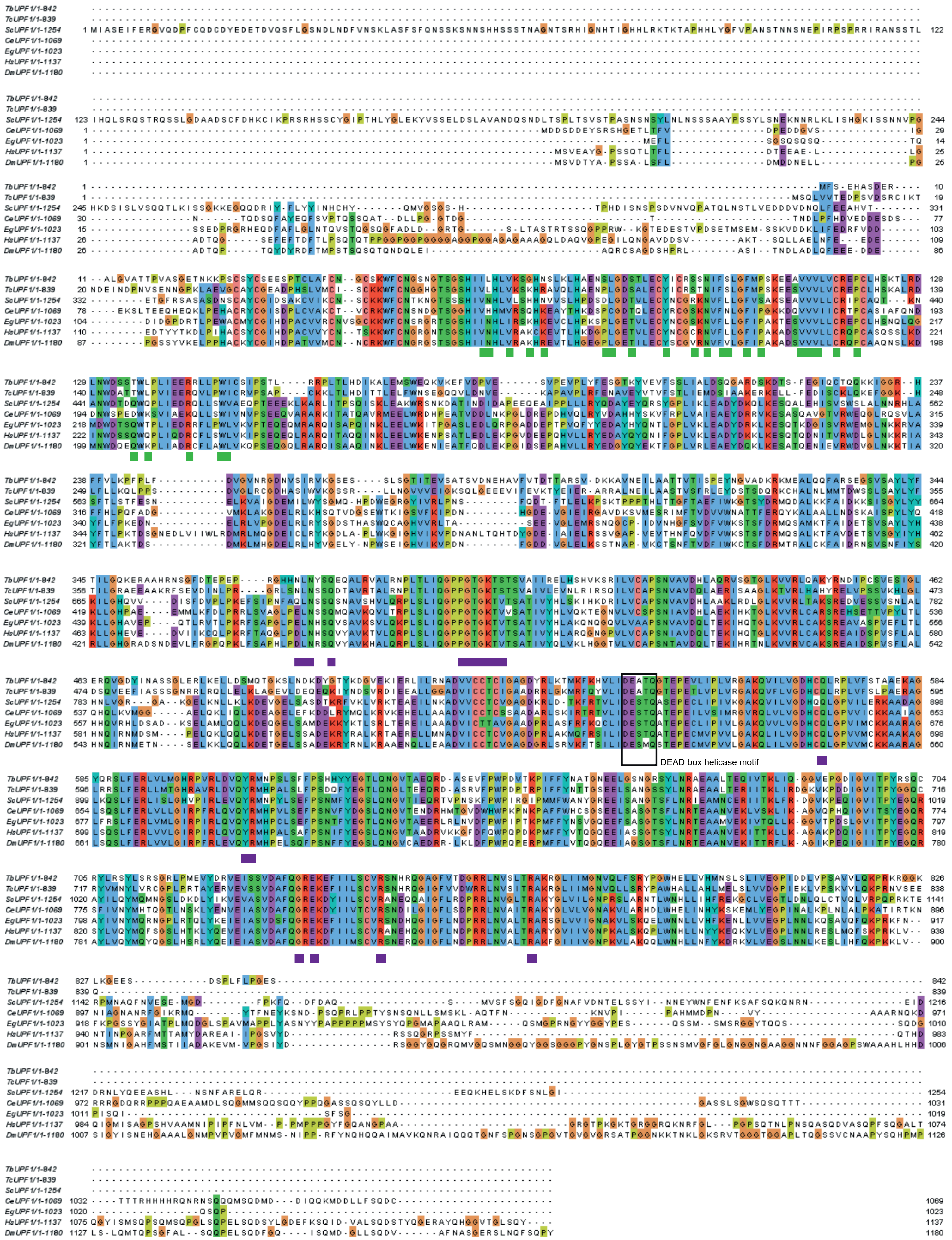

Figure S1B: UPF1 sequence comparison. Residues that are involved in UPF2 interaction in the human protein (PMID: 19556969) are indicated (green boxes below the alignment). Residues engaging in ATP binding have purple boxes. The DEAD-box helicase motif of UPF1 is highlighted. Hs, *Homo sapiens* (Q92900); Ce, *Caenorhabditis elegans* (O76512); Dm, *Drosophila melanogaster* (Q9VYS3); Eg, *Euglena gracilis* (EG_transcript_1144); Sc, *Saccharomyces cerevisiae* (P30771); Tb, *Trypanosoma brucei* (Tb927.5.2140); Tc, *Trypanosoma cruzi* (C3747_24g302).
