# Supplementary material for: Trypanosomes lack a canonical EJC but possess an UPF1 dependent NMD-like pathway
Source: PLoS One. 2025 Mar 7;20(3):e0315659. doi: 10.1371/journal.pone.0315659 (PMC11888146; doi:10.1371/journal.pone.0315659)
Supplement: S5 Fig — (PDF) [file pone.0315659.s009.pdf]

Figure S5

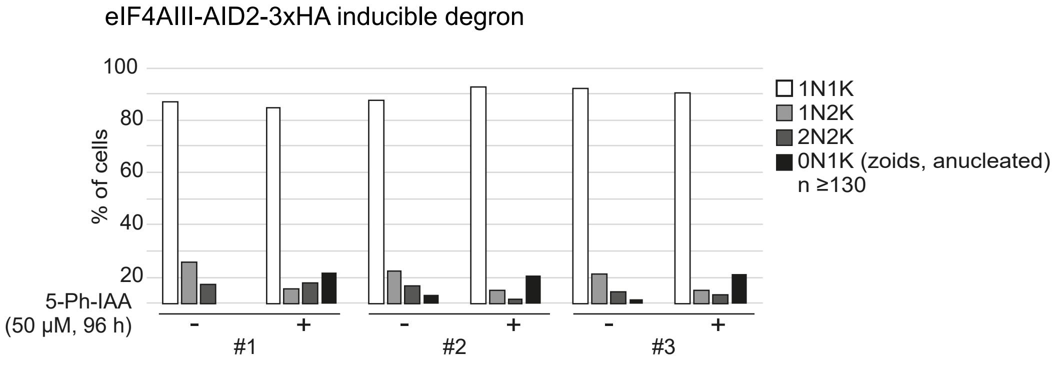

Figure S5: eIF4AIII depletion causes no specific cell cycle block
Trypanosome cells were stained with the DNA dye DAPI and cells were classified according to their cell cycle stage by counting the number of nuclei (N) and kintoplasts (K) for at least 130 cells. The kinetoplast (the visible DNA of the single mitochondrion) divides prior to the nucleus, allowing a classification into 1K1N, 2K1N and 2K2N cells. No significant differences in the distribution of the different cell cycle stages was observed upon induction of eIF4AIII depletion, apart from a small increase in anucleated cells.
