# Supplementary figures and images for: Trypanosomes lack a canonical EJC but possess an UPF1 dependent NMD-like pathway
Source: PLoS One. 2025 Mar 7;20(3):e0315659. doi: 10.1371/journal.pone.0315659 (PMC11888146; doi:10.1371/journal.pone.0315659)

Figure S6

A Y14

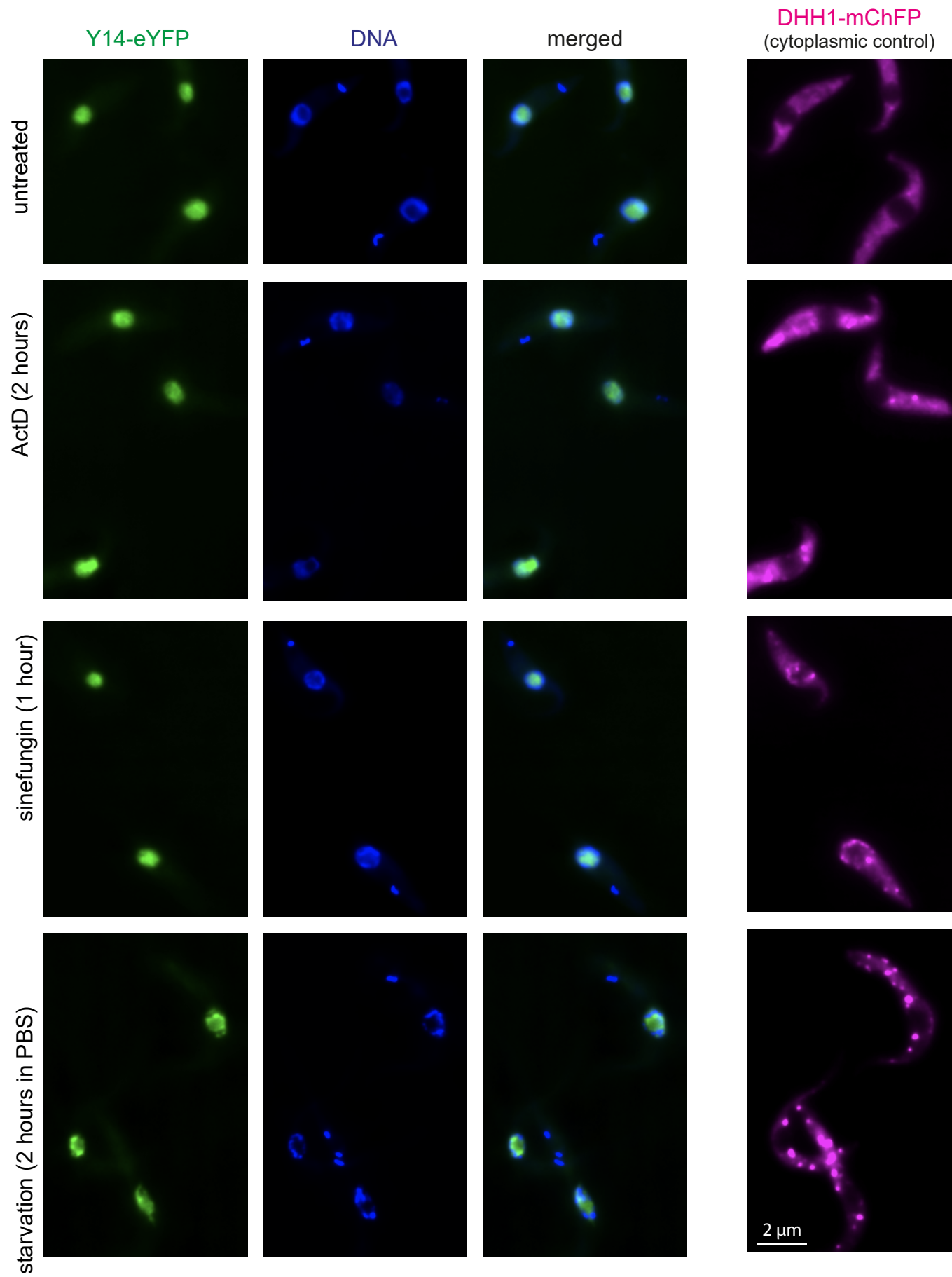

Supplement: S6A Fig — (PDF) [file pone.0315659.s010.pdf]

Figure S6

B Magoh

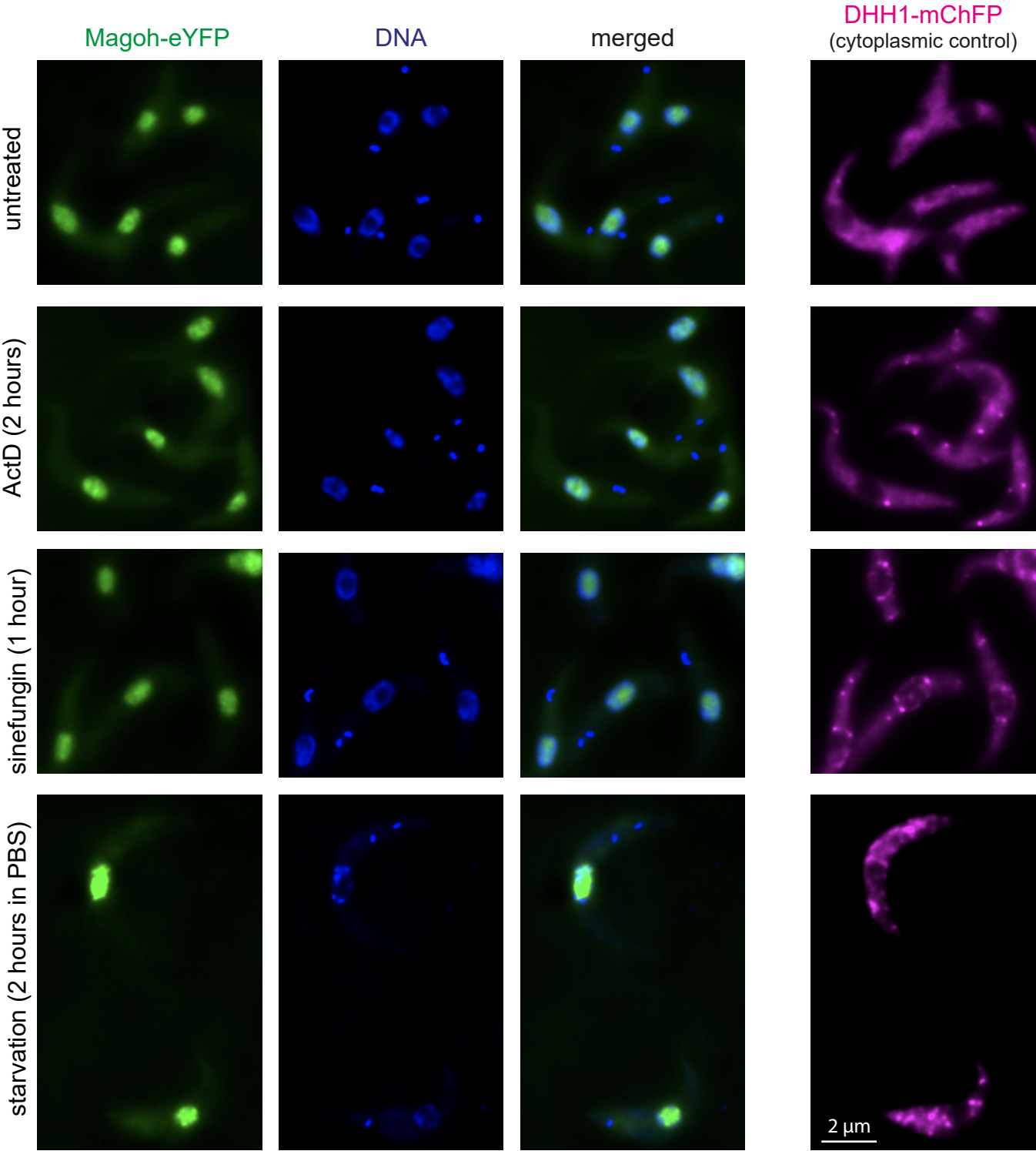

Supplement: S6B Fig — (PDF) [file pone.0315659.s011.pdf]
