# Supplementary material for: Trypanosomes lack a canonical EJC but possess an UPF1 dependent NMD-like pathway
Source: PLoS One. 2025 Mar 7;20(3):e0315659. doi: 10.1371/journal.pone.0315659 (PMC11888146; doi:10.1371/journal.pone.0315659)
Supplement: S6C Fig — (PDF) [file pone.0315659.s012.pdf]

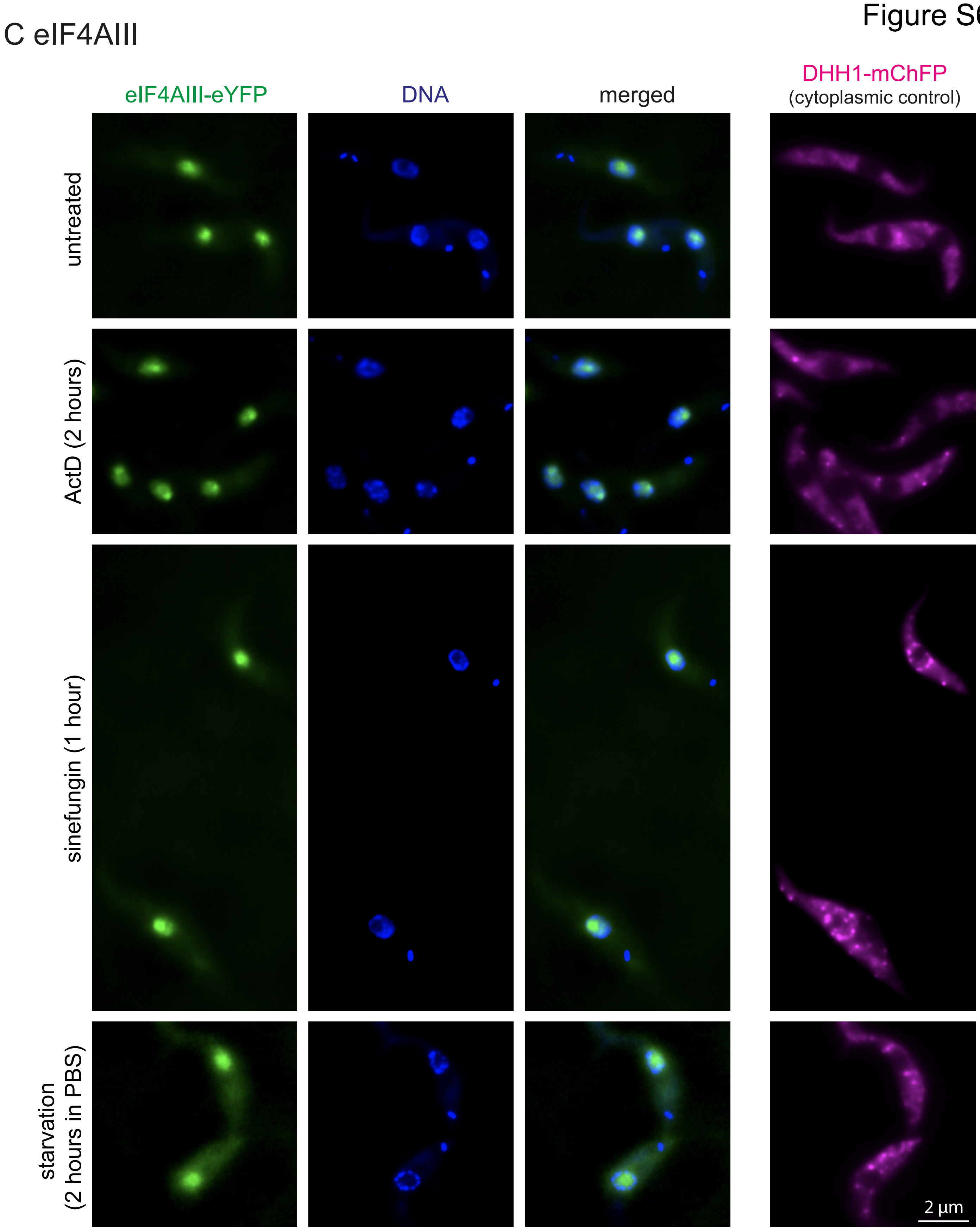

Figure S6: Cells expressing Y14 (A), Magoh (B) or eIF4AIII (C) fused to eYFP in a cell line also expressing DHH1-mChFP were treated as indicated. Projections (sum slices) of deconvolved Z-stacks (75 slices a 140 nm) are shown.
