# Supplementary material for: Trypanosomes lack a canonical EJC but possess an UPF1 dependent NMD-like pathway
Source: PLoS One. 2025 Mar 7;20(3):e0315659. doi: 10.1371/journal.pone.0315659 (PMC11888146; doi:10.1371/journal.pone.0315659)
Supplement: S7 Fig — (PDF) [file pone.0315659.s013.pdf]

Figure S7

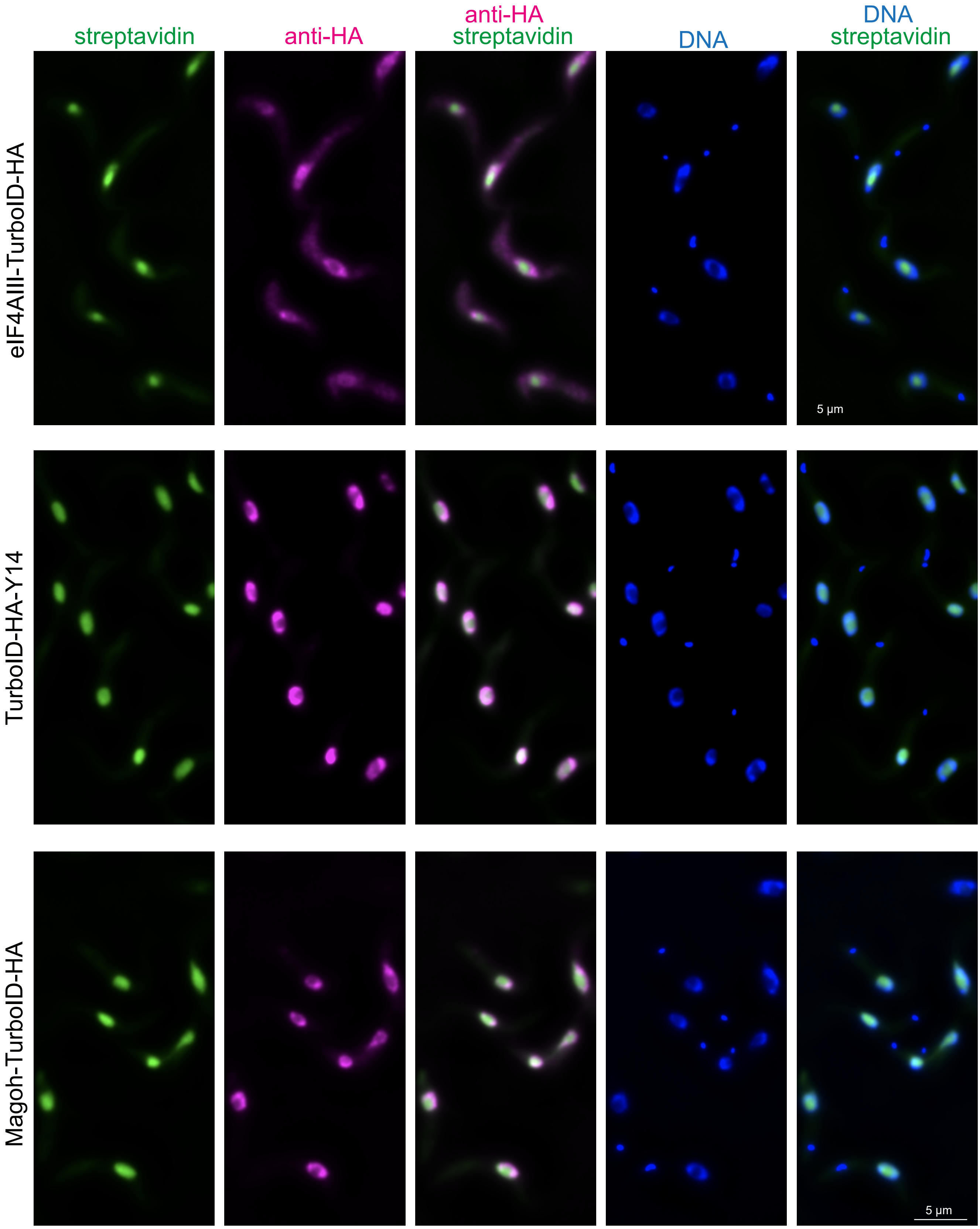

Figure S7: eIF4AIII, Y14 or Magoh were expressed fused to TurboID-HA and imaged with anti-HA (shown in pink) and streptavidin-Alexa488. Z-stack projections (sum slices) of a deconvolved Z-stack image (100 slices a 100 nm) are shown.
