# Supplementary material for: Trypanosomes lack a canonical EJC but possess an UPF1 dependent NMD-like pathway
Source: PLoS One. 2025 Mar 7;20(3):e0315659. doi: 10.1371/journal.pone.0315659 (PMC11888146; doi:10.1371/journal.pone.0315659)
Supplement: S8 Fig — (PDF) [file pone.0315659.s014.pdf]

Figure S8

A NTFL2-AID-3xHA inducible degron

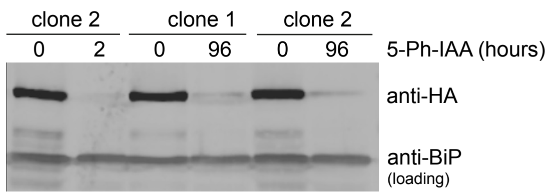

B

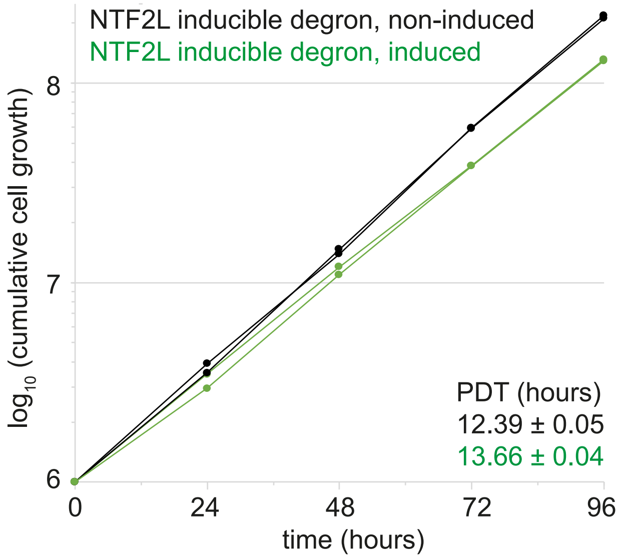

Figure S8: Two clonal cell lines (clone 1 and clone 2) were generated for the **depletion of NTFL2 via the AID2 system.**

A) Western blot loaded with cell extracts of cells incubated with 50 µM 5-Ph-IAA for 0, 2 or 96 hours. NTFL2-AID-3HA protein is detected with anti-HA. Anti-BIP served as loading control. The NTFL2 protein becomes undetectable within 2 hours of induction.

B) Growth was monitored for both clones over 96 hours, in the presence and absence of 5-Ph-IAA. The population doubling times (PDT) are only marginally increased upon induction.
