# Supplementary material for: Trypanosomes lack a canonical EJC but possess an UPF1 dependent NMD-like pathway
Source: PLoS One. 2025 Mar 7;20(3):e0315659. doi: 10.1371/journal.pone.0315659 (PMC11888146; doi:10.1371/journal.pone.0315659)
Supplement: S9 Fig — (PDF) [file pone.0315659.s015.pdf]

Figure S9

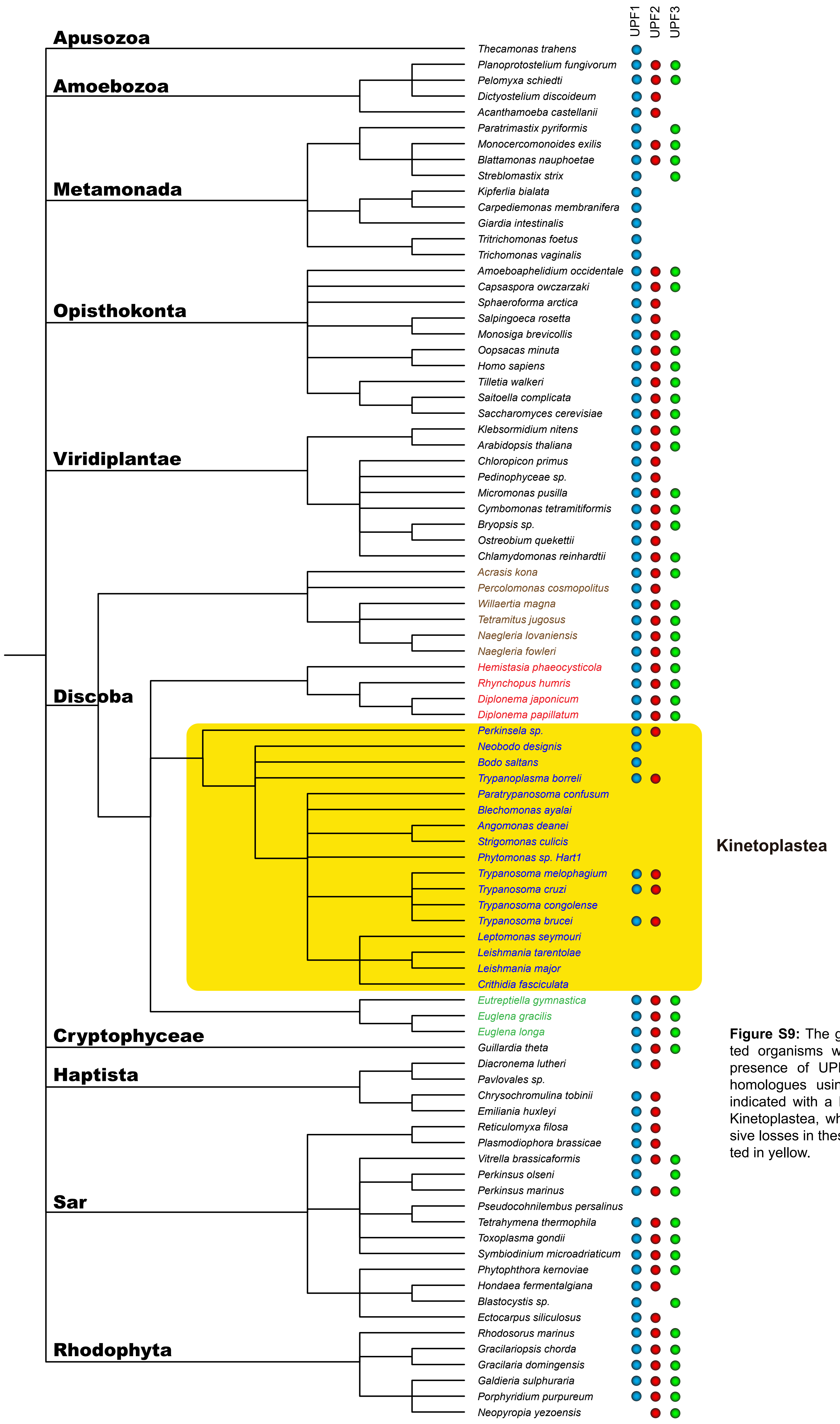

Figure S9: The genomes of the indicated organisms were screened for the presence of UPF1, UPF2 and UPF3 homologues using Blast; presence is indicated with a ball. The group of the Kinetoplastea, which experience extensive losses in these proteins, is highlighted in yellow.
