# Supplementary material for: Trypanosomes lack a canonical EJC but possess an UPF1 dependent NMD-like pathway
Source: PLoS One. 2025 Mar 7;20(3):e0315659. doi: 10.1371/journal.pone.0315659 (PMC11888146; doi:10.1371/journal.pone.0315659)

Raw data

Figure 1: eIF4AIII-inducible degradation (Figure 1B)

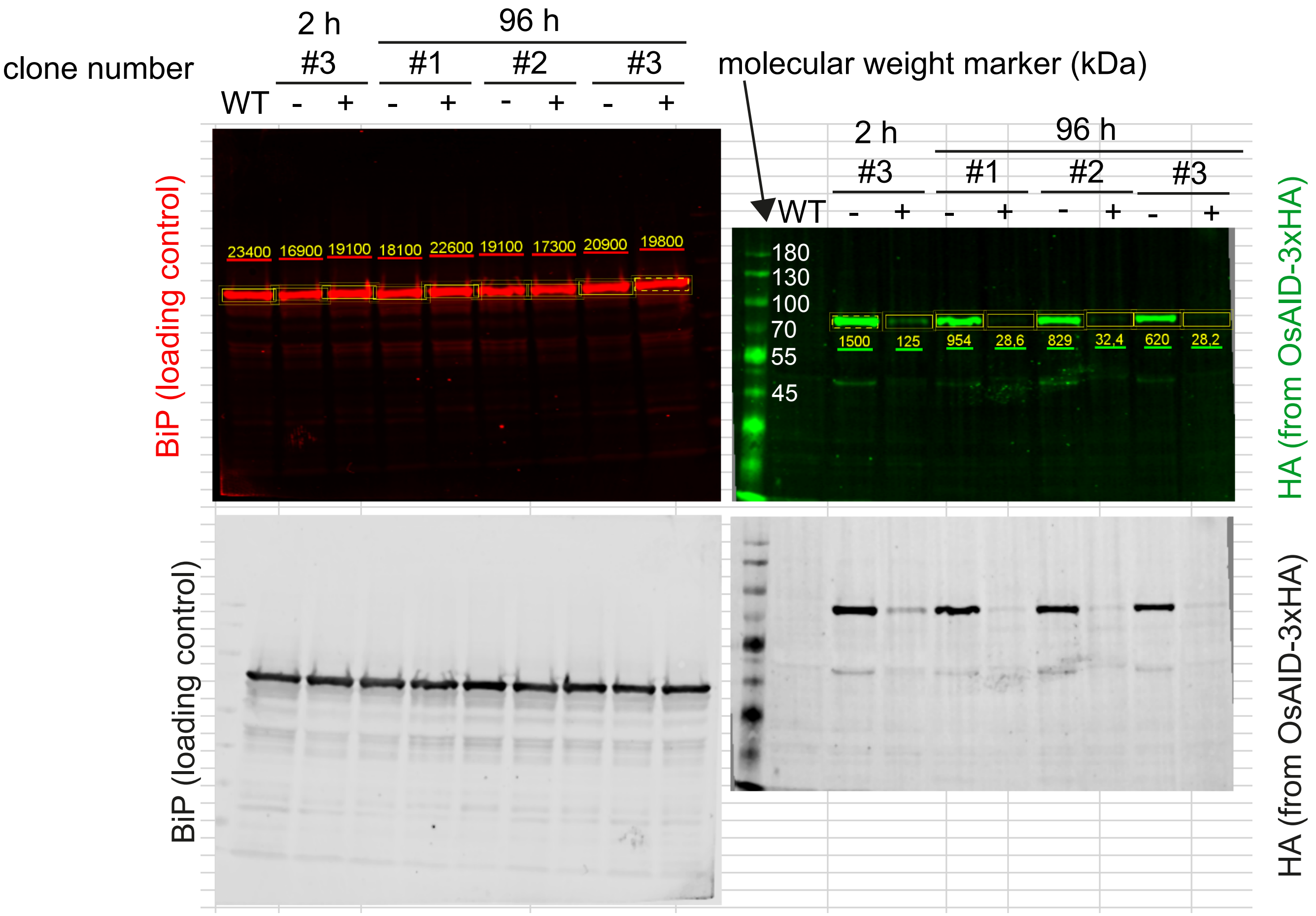

	eIF4AIII auxin quantification								
	Order								
	WT	#3	#3 2h	#1	#1 2h	#2	#2 2h	#3	#3 2h
red (BiP)	19800	20900	17300	19100	22600	18100	19100	16900	23400
green (HA)	0	1500	125	954	28.6	829	32.4	620	28.2
% HA/BiP		7.177033493	0.72254	4.994764398	0.1265487	4.58011	0.16963	3.669	0.12051
% degradation			89.9326		97.466374		96.2963		96.7151

Figure 2: UPF1 auxin-inducible degradation (Figure 4A)

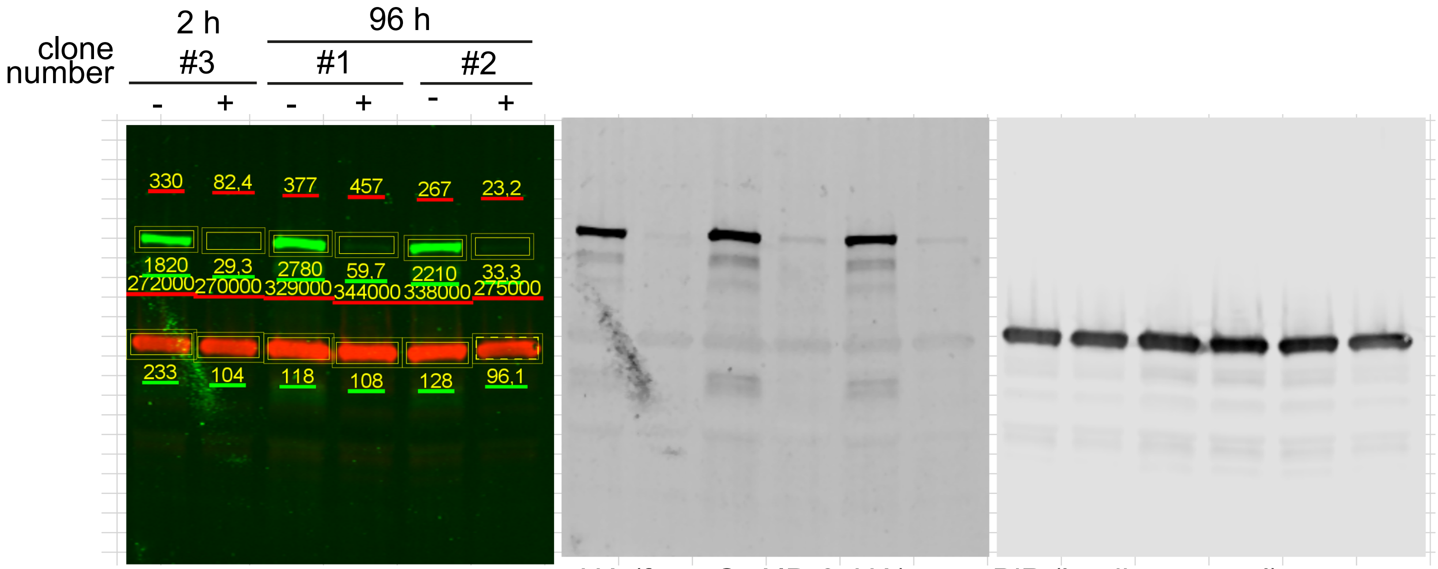

BiP (loading control)
HA (from OsAID-3xHA)

HA (from OsAID-3xHA)

BiP (loading control)

Order	#2	#2 2h	#1	#1 96h	#2	#2 96h
HA (green)	1820	29.3	2780	59.7	2210	33.3
BiP (red)	272000	270000	329000	344000	338000	275000
HA/BiP	66.91176	1.085185	84.49848	1.735465	65.38462	1.210909
		98.37818		97.94616		98.14802

Figure 3: NTF2L auxin-inducible degradation (Figure S8)

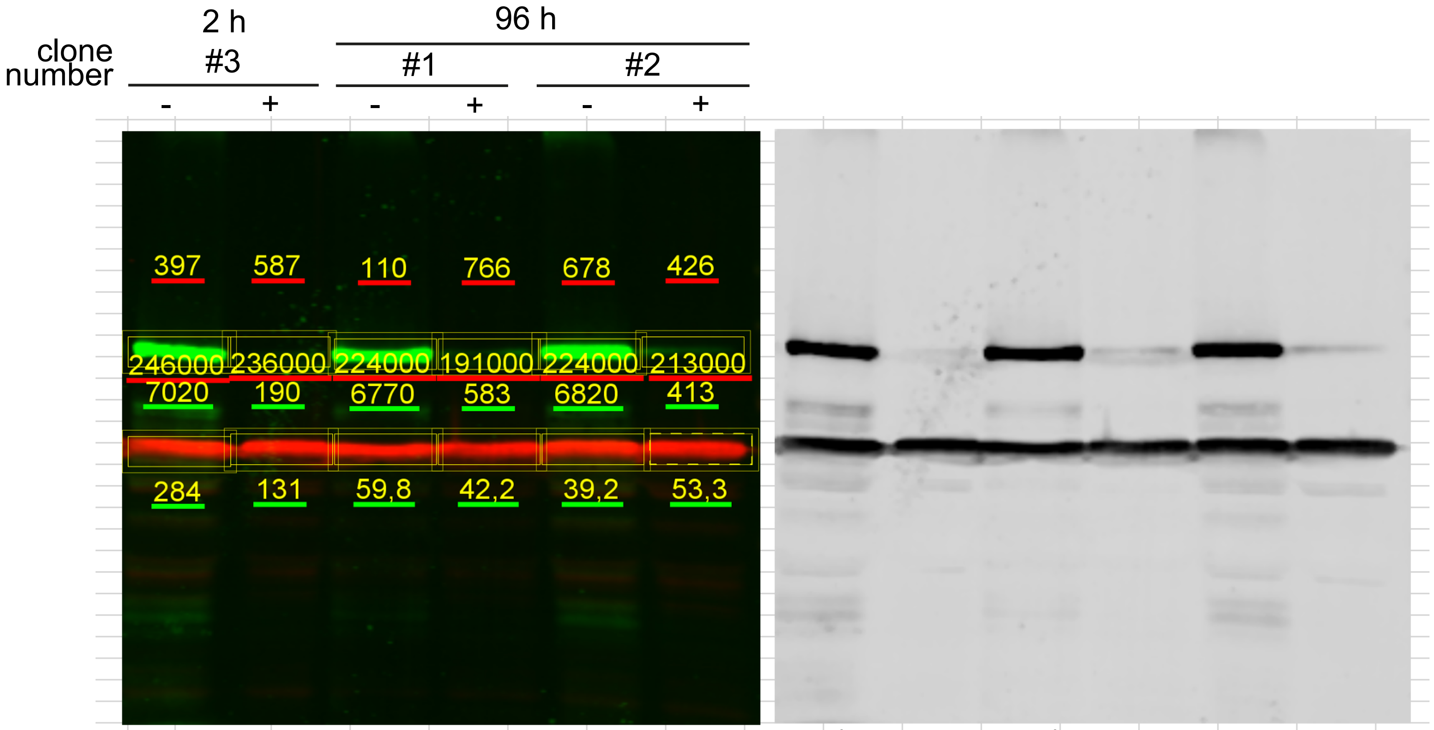

BiP (loading control)
HA (from OsAID-3xHA)

BiP (loading control)
HA (from OsAID-3xHA)

Order	#2	#2 2h	#1	#1 96h	#2	#2 96h
HA (green)	7020	190	6770	583	6820	412
BiP (red)	246000	236000	224000	191000	224000	213000
HA/BiP	285.3659	8.050847	302.2321	30.52356	304.4643	19.34272
		97.17876		89.90062		93.64696

Raw data

Figure 4: Northern blot (Figure 4E)

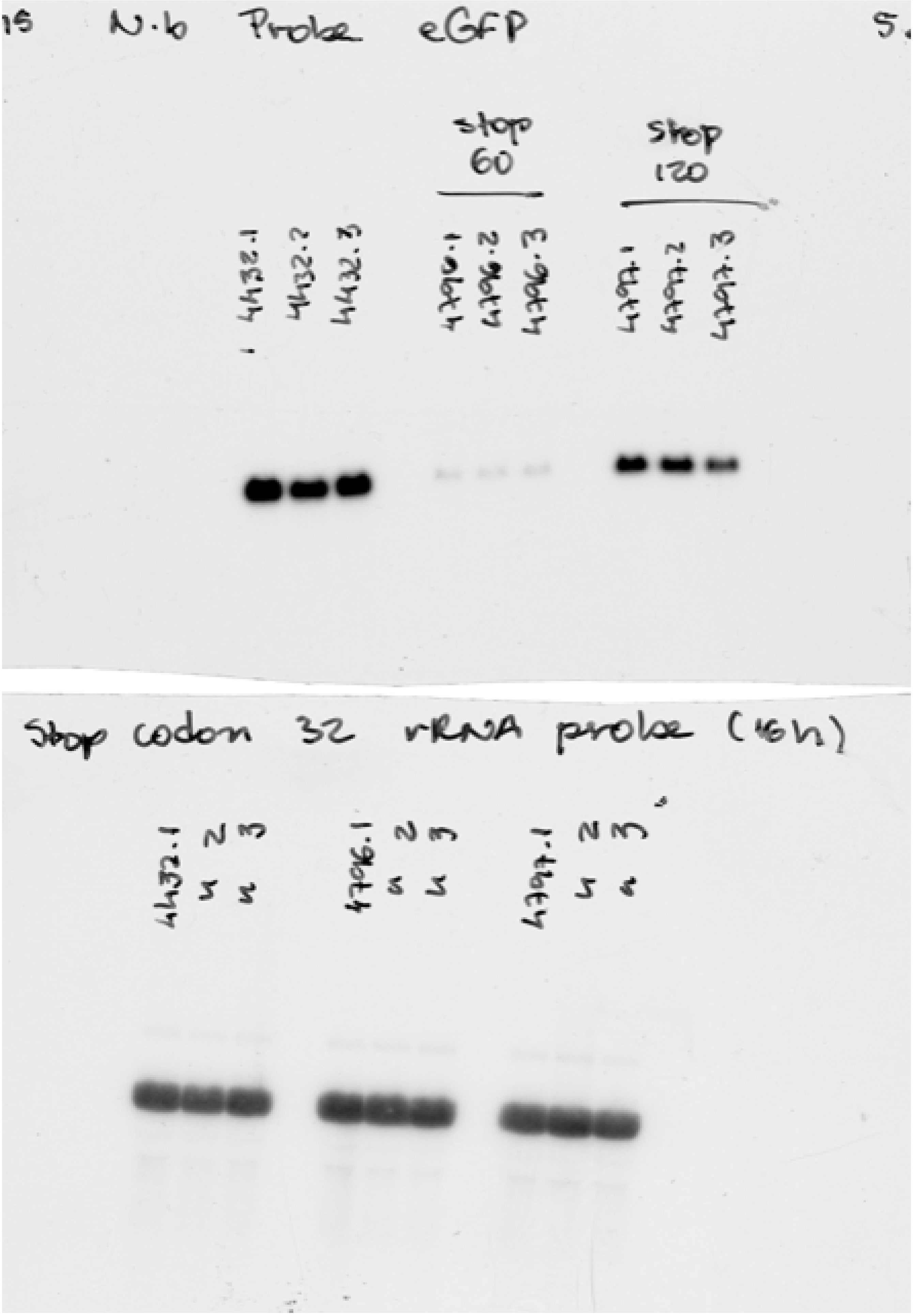

Supplement: S1 Raw Images — (PDF) [file pone.0315659.s016.pdf]
