# Supplementary material for: Trypanosomes lack a canonical EJC but possess an UPF1 dependent NMD-like pathway
Source: PLoS One. 2025 Mar 7;20(3):e0315659. doi: 10.1371/journal.pone.0315659 (PMC11888146; doi:10.1371/journal.pone.0315659)
Supplement: Attachment — Submitted filename: rebuttal_auresp_2.docx [file pone.0315659.s018.docx]

PONE-D-24-18641R1

Trypanosomes lack a canonical EJC but possess an UPF1 dependent NMD pathway

PLOS ONE

Dear Dr. Zoltner,

Thank you for submitting your manuscript to PLOS ONE. After careful consideration, we feel that it requires some minor edits to the text based on new comments from reviewers 1 and 3. Therefore, we invite you to submit a revised version of the manuscript that addresses the points raised during the review process.

The revised manuscript will not need to go out for another round of reviews and the final decision will be made at the editorial level. So, we expect a quick turnaround for decision on the next submission.

Please submit your revised manuscript by Jan 03 2025 11:59PM. If you will need more time than this to complete your revisions, please reply to this message or contact the journal office at [plosone@plos.org](mailto:plosone@plos.org). When you're ready to submit your revision, log on to https://www.editorialmanager.com/pone/ and select the 'Submissions Needing Revision' folder to locate your manuscript file.

Please include the following items when submitting your revised manuscript:

- A rebuttal letter that responds to each point raised by the academic editor and reviewer(s). You should upload this letter as a separate file labeled 'Response to Reviewers'.
- A marked-up copy of your manuscript that highlights changes made to the original version. You should upload this as a separate file labeled 'Revised Manuscript with Track Changes'.
- An unmarked version of your revised paper without tracked changes. You should upload this as a separate file labeled 'Manuscript'.

If you would like to make changes to your financial disclosure, please include your updated statement in your cover letter. Guidelines for resubmitting your figure files are available below the reviewer comments at the end of this letter.

If applicable, we recommend that you deposit your laboratory protocols in protocols.io to enhance the reproducibility of your results. Protocols.io assigns your protocol its own identifier (DOI) so that it can be cited independently in the future. For instructions see: [https://journals.plos.org/plosone/s/submission-guidelines#loc-laboratory-protocols](https://track.editorialmanager.com/CL0/https:%2F%2Fjournals.plos.org%2Fplosone%2Fs%2Fsubmission-guidelines%23loc-laboratory-protocols/1/010f019345171a3b-9027d669-dc3a-48f1-9429-6796b350de39-000000/h_kH2U_ziWp-W-O49L0g_78k3iBgX2VJw-PQ9GO-eX0=186). Additionally, PLOS ONE offers an option for publishing peer-reviewed Lab Protocol articles, which describe protocols hosted on protocols.io. Read more information on sharing protocols at [https://plos.org/protocols?utm_medium=editorial-email&utm_source=authorletters&utm_campaign=protocols](https://track.editorialmanager.com/CL0/https:%2F%2Fplos.org%2Fprotocols%3Futm_medium=editorial-email%26utm_source=authorletters%26utm_campaign=protocols/1/010f019345171a3b-9027d669-dc3a-48f1-9429-6796b350de39-000000/nOqdqEE-nMgP57QCsV7qTcllaaWM9PKNHPSKipine54=186).

We look forward to receiving your revised manuscript.

Kind regards,

Guramrit Singh
Academic Editor
PLOS ONE

Journal Requirements:

Please review your reference list to ensure that it is complete and correct. If you have cited papers that have been retracted, please include the rationale for doing so in the manuscript text, or remove these references and replace them with relevant current references. Any changes to the reference list should be mentioned in the rebuttal letter that accompanies your revised manuscript. If you need to cite a retracted article, indicate the article’s retracted status in the References list and also include a citation and full reference for the retraction notice.


[Note: HTML markup is below. Please do not edit.]

Reviewers' comments:

Reviewer's Responses to Questions

**Comments to the Author**

1. If the authors have adequately addressed your comments raised in a previous round of review and you feel that this manuscript is now acceptable for publication, you may indicate that here to bypass the “Comments to the Author” section, enter your conflict of interest statement in the “Confidential to Editor” section, and submit your "Accept" recommendation.

Reviewer #1: All comments have been addressed

Reviewer #2: All comments have been addressed

Reviewer #3: (No Response)

2. Is the manuscript technically sound, and do the data support the conclusions?

The manuscript must describe a technically sound piece of scientific research with data that supports the conclusions. Experiments must have been conducted rigorously, with appropriate controls, replication, and sample sizes. The conclusions must be drawn appropriately based on the data presented.

Reviewer #1: Yes

Reviewer #2: Yes

Reviewer #3: Yes

3. Has the statistical analysis been performed appropriately and rigorously?

Reviewer #1: Yes

Reviewer #2: Yes

Reviewer #3: Yes

4. Have the authors made all data underlying the findings in their manuscript fully available?

The [PLOS Data policy](http://track.editorialmanager.com/CL0/http:%2F%2Fwww.plosone.org%2Fstatic%2Fpolicies.action%23sharing/1/010f019345171a3b-9027d669-dc3a-48f1-9429-6796b350de39-000000/sr80eY8ipvFllcpzfZ7-X-69JzJ8HLUMBsv3qYE3-l4=186) requires authors to make all data underlying the findings described in their manuscript fully available without restriction, with rare exception (please refer to the Data Availability Statement in the manuscript PDF file). The data should be provided as part of the manuscript or its supporting information, or deposited to a public repository. For example, in addition to summary statistics, the data points behind means, medians and variance measures should be available. If there are restrictions on publicly sharing data—e.g. participant privacy or use of data from a third party—those must be specified.

Reviewer #1: Yes

Reviewer #2: Yes

Reviewer #3: Yes

5. Is the manuscript presented in an intelligible fashion and written in standard English?

PLOS ONE does not copyedit accepted manuscripts, so the language in submitted articles must be clear, correct, and unambiguous. Any typographical or grammatical errors should be corrected at revision, so please note any specific errors here.

Reviewer #1: Yes

Reviewer #2: Yes

Reviewer #3: Yes

6. Review Comments to the Author

Please use the space provided to explain your answers to the questions above. You may also include additional comments for the author, including concerns about dual publication, research ethics, or publication ethics. (Please upload your review as an attachment if it exceeds 20,000 characters)

Reviewer #1: The authors have adequately addressed all previous comments from my side, either by including new data or by proper explanation. I have no further major comments and recommend publication of this manuscript.

Nevertheless, I would like to use the opportunity to comment on the added NMD reporter data:
I am particularly pleased that the new experiments using an eGFP-based NMD reporter were insightful and helped to clarify the "NMD capacity" of trypanosomes. It seems that trypanosomes are in principle able to degrade PTC-containing mRNAs in an UPF1-dependent manner. The clear distinction whether the degradation is due to "canonical NMD" or other UPF1- and translation-dependent processes is not trivial and would require more detailed investigation (not in this manuscript though). One could even argue how NMD would be defined under these circumstances (e.g. does it require UPF2? Is the helicase activity of UPF1 required? Etc.). In light of these open questions and since the NMD(-like) activity seems to be only relevant for reporter mRNAs, the authors may want to soften some strong statements about the presence of an NMD pathway (e.g. in the new title).

We agree that there are several open questions remaining about the precise role of UPF1 in the degradation of PTC-containing mRNAs and thus, overall, the definition of the NMD pathway in trypanosomes (and likewise other unicellular organisms such as yeast). We therefore have softened the title by referring to an NMD-like pathway:

*‘Trypanosomes lack a canonical EJC but possess an UPF1 dependent NMD-like pathway’*

Same in the discussion:

*Notably, UPF1 deletion in other organisms with an EJC-independent NMD, such as S. pombe, also does not completely restore the steady state levels of early PTC reporters (85). We thus conclude that there is an EJC-independent NMD-like pathway in T. brucei which significantly relies on UPF1.*

*In conclusion, we provide evidence for the presence of an EJC independent NMD-like pathway in T. brucei that is fully or partially dependent on UPF1.*

Further, we have added more discussion of this matter (see below, response to R3) and compare our findings critically to previous observations.

A technical detail I wanted to comment on:
If I understand it correctly, the UPF1 depletion was performed for 2 hours before harvesting and extracting the RNA. I am not familiar with transcription kinetics in trypanosomes, but I was simply wondering whether this timeframe was maybe too short to see the "full" rescue of the early (position 60) PTC reporter? I certainly do not ask for those experiments to be repeated, but it could be another point for discussing the apparently incomplete rescue.

Yes correct – we chose 2 hours depletion to restrict secondary effects of the auxin depletion. This was working for the early PTC (with abundance almost doubling, although levels were not fully restored). Simultaneously, we were clearly observing a general effect on the PTC-independent transcript abundance (which we coin to the global loss of fitness upon depletion). Due to the latter effect, we refrained from testing longer depletion times. We agree that residual transcripts made before the onset of full UPF1 depletion (and not completely turned over) may be responsible for the incomplete rescue and added the following to the discussion:

*Another potential factor obscuring the rescue is the residual abundance of transcripts formed before complete UPF1 depletion during the 2h induction time, chosen to restrict secondary effects. Nevertheless, our data show that UPF1 contributes to NMD, but they do not fully rule out the possibility that additional factors contribute.*

Reviewer #2: The authors have adequately addressed the comments by the reviwers in the revised version of the manuscript. The manuscript is improved and included new results to base their conclusions. Therefore, have no further comments.

Reviewer #3: The authors have addressed my comments and also introduced interesting new observations which change some of the conclusions. They are however going to have to revise this manuscript because the Abstract is 373 words long but the guidelines restrict it to 300 words.

Thanks for spotting – we shortened the abstract.

Because of some newly-introduced parts, there is also a small number of new issues to address:

If there really is UPF-dependent NMD the authors need to address, at least very briefly,
(a) how this is consistent with the results in ref 48 and

We see the data interpretation in Ref 48 (PMID: 34541528; cited in the introduction) as oversimplified for the reasons listed below.

*1. NMD is not that efficient, so there will be some mRNA, even in humans 25% avoids turnover*

*2. The effectiveness of NMD varies from one mRNA to another,*

*3. Where there is a frame shift (first example figure 6 in the paper) no account is taken of the consequent change in codon use*

*4. The allocation of reads cannot be that accurate, looking at the second example in Figure 6 the abundance (RPM) in regions 4 and 5 are reversed. This is difficult to explain if the measurements are accurate. It would have been better to sum the two sets of allele specific RNAseq reads*

*5. There are some examples in figures S9 and S10 where the PTC allele is less abundant.*

*6. Some of the mRNAs chosen are developmentally regulated, which may have an effect*

However, we feel that we are not in position (and don’t intend) to attack these results as our data is restricted to a reporter gene. For this, we show that PTCs reduce transcript levels in a position specific manner (Northern blot; Fig 4E) and that the drop in early PTC transcripts can be partly rescued by UPF-1 depletion (Fig 4F).

(b) how it might be triggered, given the presence of some very long 3'-UTRs in Kinetoplastid mRNAs and the fact that previous reporter experiments (ref 46) were difficult to interpret. (I realise that the answer to the latter might be "we have no idea".)

Thanks, we have added a sentence to the discussion.

Given that abnormally long 3'-UTRs contribute to NMD triggering (88) a further open question how NMD is mediated in the presence of long 3'-UTRs present in many trypanosome genes (46).

Minor text issues (just sound awkward)

The section about TREX is confusing because it is now internally contradictory. I suggest starting with "Trypanosomes lack obvious homologues of most proteins of the TREX..." instead of "Trypanosomes lack homologues of most proteins of the TREX..."

Thanks. Done.

"Consistent, UPF1 depletion has no major phenotype.." needs to be changed - "Consistent with this, UPF1-depleted cells have an almost normal phenotype" ("Consistent" can't be used on its own, and ll cells have a phenotype.....)

Agree. Changed.

"As a consequence, the presence of a canonical exon junction complex, as well as an NMD pathway have become redundant and are in the process of being lost." - I suggest instead "As a consequence, the canonical exon junction complex and the NMD pathway have become redundant and are in the process of being lost." ("Superfluous" might also be better than "redundantP>

Thanks. Done.

7. PLOS authors have the option to publish the peer review history of their article ([what does this mean?](https://track.editorialmanager.com/CL0/https:%2F%2Fjournals.plos.org%2Fplosone%2Fs%2Feditorial-and-peer-review-process%23loc-peer-review-history/1/010f019345171a3b-9027d669-dc3a-48f1-9429-6796b350de39-000000/iuthlsdU-I6Mei109ej2vWoyRb1VMkZl27vwSXpQ8es=186)). If published, this will include your full peer review and any attached files.


If you choose “no”, your identity will remain anonymous but your review may still be made public.


**Do you want your identity to be public for this peer review?** For information about this choice, including consent withdrawal, please see our [Privacy Policy](https://track.editorialmanager.com/CL0/https:%2F%2Fwww.plos.org%2Fprivacy-policy/1/010f019345171a3b-9027d669-dc3a-48f1-9429-6796b350de39-000000/Bgmveu_W6gCC800KAC7rhFGRN3rVcFxd7sqXAqQ2k5I=186).

Reviewer #1: **Yes:** Volker Boehm

Reviewer #2: No

Reviewer #3: No

[NOTE: If reviewer comments were submitted as an attachment file, they will be attached to this email and accessible via the submission site. Please log into your account, locate the manuscript record, and check for the action link "View Attachments". If this link does not appear, there are no attachment files.]

While revising your submission, please upload your figure files to the Preflight Analysis and Conversion Engine (PACE) digital diagnostic tool, [https://pacev2.apexcovantage.com/](https://track.editorialmanager.com/CL0/https:%2F%2Fpacev2.apexcovantage.com%2F/1/010f019345171a3b-9027d669-dc3a-48f1-9429-6796b350de39-000000/w76xrzSTrVIIqBnXAly5buf5DssqODgaPHIx460vByg=186). PACE helps ensure that figures meet PLOS requirements. To use PACE, you must first register as a user. Registration is free. Then, login and navigate to the UPLOAD tab, where you will find detailed instructions on how to use the tool. If you encounter any issues or have any questions when using PACE, please email PLOS at [figures@plos.org](mailto:figures@plos.org). Please note that Supporting Information files do not need this step.
